# Anti-stigma campaign: the COVID-19 stigma and associated factors among Chinese young students

**DOI:** 10.3389/fpubh.2025.1596929

**Published:** 2025-07-28

**Authors:** Cong Wang, Yun-Fei Mu, Jia Cai, Yu Wang, Zhong-Yue Deng, Ai-Ping Deng, Hong-Jun Song, Tian-Ming Zhang, Xin-Yi Zhao, Yi-Yue Yang, Li Yin, Yi Huang, Jian-Jun Luo, Mao-Sheng Ran

**Affiliations:** ^1^Mental Health Center, West China Hospital, Sichuan University, Chengdu, Sichuan, China; ^2^Department of Social Psychiatry, West China Hospital, Sichuan University, Chengdu, Sichuan, China; ^3^West China School of Nursing, Sichuan University, Chengdu, Sichuan, China; ^4^Department of Social Work, Shanghai University, Shanghai, China; ^5^School of Health Humanities, Peking University, Beijing, China; ^6^Chongqing Mental Health Center, Chongqing, Jiangbei, China

**Keywords:** COVID related stigma, youth, students, China, influence factors

## Abstract

**Introduction:**

COVID-19-related stigma, a persistent consequence of the pandemic, poses a significant threat to the well-being of individuals, particularly young students in crucial developmental stages. This study aimed to investigate the prevalence of stigma views towards COVID-19 patients among young students in Sichuan Province, China, following the lifting of pandemic restrictions, and explored the associated influencing factors.

**Methods:**

A cross-sectional survey was conducted online involving 82,873 young students. Data on demographic information, COVID-19-related experiences, and stigma views were collected. Logistic regression analysis identified risk factors for stigma, while Random Forest (RF) modeling ranked the importance of these factors.

**Results:**

20,155 (24.3%) participants reported stigmatizing views toward COVID-19 patients. Higher levels of psychological stress, particularly post-restriction lifting, significant emerged as a risk factor for stigma. Both self-infection and family members' infection were risk factors of stigma, although psychological stress proved more influential. Furthermore, male students, those from minority groups, and those with rural household registrations exhibited higher stigma risk.

**Discussion:**

These findings highlight the enduring nature of COVID-19 stigma among young students even after restrictions lifted. Targeted interventions aimed at reducing stigma and mitigating psychological stress are crucial for supporting the well-being of this vulnerable population.

## 1 Introduction

Stigma is the phenomenon that ascribing certain attributes of individuals as discrediting, resulting in the general person being discounted or devalued (1). Stigma may have significant impacts on resource acquisition, social interactions, physical and mental health, and overall quality of life (2). In the context of health, stigma is associated with certain health conditions (e.g., leprosy, epilepsy, cancer, mental illness, and infectious disease, overweigh or obesity) (2–5).

Stigma always accompanied the public health emergencies, such as Ebola virus disease (EVD), severe acute respiratory syndrome (SARS) (6). At the end of 2019, the infectious disease caused by the novel coronavirus first appeared, and the World Health Organization confirmed the infectious disease as the 2019 novel coronavirus (COVID-19) pandemic in March 2020 (7). The COVID-19 pandemic had affected almost every aspect of people's lives, including physical activity, sleep, time use and mental health (8). Besides the threat to public physical and mental health, the pandemic had caused secondary disaster, such as COVID-19 stigma (9). A previous study conducted during the early stages of the COVID-19 pandemic had found that 90% of the respondents inside of China reported stigma attitude toward people infected (10).

COVID-19 patients and survivors, Asian group, those with a travel history to affected countries, healthcare workers, and even close contacts and suspected cases were targets of COVID-19 stigma (11, 12). COVID-19 stigma exposed people to more harmful outcome than infection (13, 14). People who were stigmatized may experience verbal abuse, violent attacks, unjustifiable fired, obstacles to promotion, difficulties in renting, social avoidance, and even being denied entry to public places (15–17). Moreover, people would delay medical treatment in order to avoid stigma, which may lead adverse effect on their physical health and hinder virus prevention and control efforts (6, 10, 11). Stigma would also threaten the mental well being of those related to COVID-19 (14, 18). Even for individuals without infection, COVID-19 stigma was significantly associated with depression, anxiety and post-traumatic stress disorder (PTSD) (19).

Previous studies focused on the COVID-19 stigma of ethnic minorities, healthcare workers, frontline workers, COVID-19 patients and survivors, as well as their families. However, few studies focused on the COVID-19 stigma among adolescent and youth students. The pandemic could be divided into three stages in China: (1) the national lockdown phase: January 1, 2020 to April, 2020; (2) the regular prevention and control phase: April 30, 2022 to December 6, 2022; and (3) the phase after the restrictions lifted: after December 7, 2022 (20). People perceived different levels of infection risks in three stages, and the stigma attitudes toward COVID-19 patients varied accordingly. However, most previous studies of stigma have been conducted during the first two stages. Few studies on stigma related to patients with COVID-19 infection among adolescent and youth students have been conducted after the lifting of COVID-19 restrictions. Thus, this study aimed to explore the stigma related to patients with COVID-19 infection and the influencing factors of the stigma among adolescent and youth students.

## 2 Method

### 2.1 Study design and participants

This cross-sectional survey was conducted among middle and high schools, colleges and universities students in Sichuan province, China from December 14th, 2022 to February 28th, 2023. A self-designed online survey was distributed via Wenjuanxing platform. To enhance data quality, a structured approach was adopted whereby teachers distributed the questionnaires and organized students to complete them either on their mobile phones/computers or in computer classrooms. This approach effectively minimized potential obstacles and strengthened the reliability and representativeness of the findings.

In this study, participants were recruited using the convenience sampling method. A total of students from 162 schools in Sichuan Province were included, specifically 114 junior and senior high schools, and 48 colleges and universities. The questionnaires were distributed to school teachers, who then organized the students to fill them out on mobile phones or computers. For students who had difficulties using mobile phones or computers, the schools arranged for them to complete the questionnaire filling in the computer classrooms. This approach ensured the efficiency of the data collection process and guaranteed that all participants could participate smoothly. This study was conducted after the lifting of China's pandemic lockdown measures. Prior to this, Chinese students had experienced 2–3 years of home-based learning, and almost all of them had acquired the basic conditions for online learning. As a result, the issue of device usage had relatively little impact on data collection. A total of 90,118 questionnaires were collected. After strict screening, 82,873 valid questionnaires were obtained, with a response rate as high as 91.96%. This data fully demonstrates the effectiveness and reliability of the survey implementation. Participants who completed the questionnaire were included in this study. Informed consent was obtained before survey completion. Data confidentiality was ensured and the study was approved by the Ethics Committee of West China Hospital, Sichuan University (NO. 2022-1970).

### 2.2 Measurement

The self-report questionnaire consisted of four parts. The first part collected personal information and family background, including gender, ethnicity (Han Chinese, Ethnic Minority), grade level (middle school, high school, college and university), parental educational background, monthly family income, and other relevant variables. The second part focused on COVID-19-related information, such as the infection status of participants and their family members (Yes or No), quarantine experience (Yes or No), vaccination history (None, One dose, Two or more doses) and willingness (Yes or No), and pandemic's impact on students' daily life routine and academic performance (Not influenced, Partly influenced, or Seriously influenced). Psychological stress at different stages was measured using a self-reported question (0 = “No stress”, 10 = “Extreme stress”), with higher scores indicating greater stress severity. The third part assessed behavioral habits, including physical exercise, smoking and drinking. The final part evaluated stigma related to patients with COVID-19 infection.

Stigma related to patients with COVID-19 infection was assessed by the Stigma Scale formed from public stigma toward patients with mental illness (21) and recovered SARS patients (22). The Stigma Scale had been validated for Chinese adolescent students in a previous study (23). It consists of 11- items, each scored from 1 to 4 (1 = strongly disagree, 4 = strongly agree). Higher scores indicate a higher level of stigma attitude. An average score >2.5 was associated with stigma attitude toward patients, while scores ≤ 2.5 were considered no stigma (23).

### 2.3 Statistical analysis

Participants were divided into two groups according to their average scores of the Stigma Scale. Participants without stigma were included in Group 1, and participants with stigma were included in Group 2. Descriptive analysis and chi-square test were used to explore differences of general information and COVID-19-related variables between the two groups. Logistic regression was applied to identify potential influencing factors for stigma, including all significant variables from bivariate analysis in the model, which passed the Hosmer-Lemeshow goodness-of-fit test. Odds ratios and 95% confidence intervals were used to assess the strength of the independent variables and stigma, with statistical significance set at *P* < 0.05. These analyses were conducted on SPSS 27.

In addition, Random Forest, a machine learning algorithm, was used to explore the order of contributing factors to stigma. Random Forest is suitable for handling large datasets and high-dimensional variables, and it can also assess the importance of variables (24, 25). The area under the curve (AUC) of receiver operating characteristic (ROC) was used to evaluate the performance of the Random Forest (RF) model. The RF analysis was conducted via MATLAB.

## 3 Results

### 3.1 General information

As was shown in Table 1, totally 82,873 students participated in this study. The majority of the participants were female (57.3%), Han Chinese (88.7%), and high school students (43.6%). 33,314 (40.2%) participants were infected with COVID-19, and 36,402 (43.9%) participants reported their family member infected with COVID-19. The percentages of higher level of psychological stress were 26.2% during the national lockdown phase, 24.5% during the regular prevention and control phase, and 15.1% after the restriction lifted.

**Table 1:** Participants' stigma views and demographic information.

**Participants' stigma views and demographic information**	**Total**	**Without stigma**	**With stigma**	**χ^2^**	***P***
		**62,718 (75.7)**	**20,155 (24.3)**		
**Gender**				728.619	<0.001
Male	35,386 (42.7)	25,131 (40.1)	10,255 (50.9)		
Female	47,487 (57.3)	37,587 (59.9)	9,900 (49.1)		
**Ethnic**				230.200	<0.001
Han	73,537 (88.7)	56,245 (89.7)	17,292 (85.8)		
Ethnic Minority	9,336 (11.3)	6,473 (10.3)	2,863 (14.2)		
**BMI**				188.714	<0.001
Normal	53,963 (65.1)	41,470 (66.1)	12,493 (62.0)		
Overweight	7,219 (8.7)	5,577 (8.9)	1,642 (8.1)		
Obesity	21,691 (26.2)	15,671 (25.0)	6,020 (29.9)		
**Household register**				292.034	<0.001
Rural	67,740 (81.7)	50,450 (80.4)	17,290 (85.8)		
Urban	15,133 (18.3)	12,268 (19.6)	2,865 (14.2)		
**Grade**				106.067	<0.001
Middle school	24,157 (29.1)	18,085 (28.8)	6,072 (30.1)		
High school	36,111 (43.6)	26,960 (43.0)	9,151 (45.4)		
College and university	22,605 (27.3)	17,673 (28.2)	4,932 (24.5)		
Single child	20,471 (24.7)	15,657 (25.0)	4,814 (23.9)	9.552	0.002
**Monthly family income**				74.260	<0.001
≤4,999	48,451 (58.5)	36,170 (57.7)	12,281 (60.9)		
5,000–19,999	30,699 (37.0)	23,744 (37.9)	6,955 (34.5)		
≥20,000	3,723 (4.5)	2,804 (4.5)	919 (4.6)		
**Father's education background**				216.539	<0.001
Primary school and below	23,084 (27.9)	16,886 (26.9)	6,198 (30.8)		
Middle school	37,477 (45.2)	28,254 (45.0)	9,223 (45.8)		
High school	14,958 (18.0)	11,657 (18.6)	3,301 (16.4)		
College and above	7,354 (8.9)	5,921 (9.4)	1,433 (7.1)		
**Mother's education background**				153.913	<0.001
Primary school and below	30,825 (37.2)	22,777 (36.3)	8,048 (39.9)		
Middle school	33,251 (40.1)	25,205 (40.2)	8,046 (39.9)		
High school	12,782 (15.4)	9,884 (15.8)	2,898 (14.4)		
College and above	6,015 (7.3)	4,852 (7.7)	1,163 (5.8)		
Self-infected (confirmed or suspected)	33,314 (40.2)	26,937 (42.9)	6,377 (31.6)	811.584	<0.001
Being frontline worker	4,173 (5.0)	3,309 (5.3)	864 (4.3)	31.214	<0.001
Family members infected (confirmed or suspected)	36,402 (43.9)	29,533 (47.1)	6,869 (34.1)	1,047.805	<0.001
Family members had being frontline workers	2,980 (3.6)	2,417 (3.9)	563 (2.8)	49.478	<0.001
**Vaccination**				27.090	<0.001
None	492 (0.6)	335 (0.5)	157 (0.8)		
One does	896 (1.1)	635 (1.0)	261 (1.3)		
Two or more doses	81,485 (98.3)	61,748 (98.5)	19,737 (97.9)		
Willingness to vaccinate during the COVID-19	77,520 (93.5)	59,052 (94.2)	18,468 (91.6)	160.943	<0.001
Willingness to vaccinate after the restriction lifted	68,324 (82.4)	52,444 (83.6)	15,880 (78.8)	245.788	<0.001
**Psychological stress level during the national lockdown phase**				233.473	<0.001
Lower	61,158 (73.8)	47,114 (75.1)	14,044 (69.7)		
Higher	21,715 (26.2)	15,604 (24.9)	6,111 (30.3)		
**Psychological stress level during the regular prevention and control phase**				331.036	<0.001
Lower	62,590 (75.5)	48,334 (77.1)	14,256 (70.7)		
Higher	20,283 (24.5)	14,384 (22.9)	5,899 (29.3)		
**Psychological stress level after the restriction lifted**				1,160.130	<0.001
Lower	70,327 (84.9)	54,731 (87.3)	15,596 (77.4)		
Higher	12,546 (15.1)	7,987 (12.7)	4,559 (22.6)		
Quarantine	24,234 (29.2)	18,453 (29.4)	5,781 (28.7)	1,047.805	<0.001
Exercising (nearly a year)	43,819 (52.9)	31,780 (50.7)	12,039 (59.7)	502.568	<0.001
**Daily life routine**				473.600	<0.001
Not influenced	35,893 (43.3)	26,014 (41.5)	9,879 (49.0)		
Partly influenced	40,519 (48.9)	32,008 (51.0)	8,511 (42.2)		
Seriously influenced	6,461 (7.8)	4,696 (7.5)	1,765 (8.8)		
**Academic performance**				1,056.094	<0.001
Progress	15,415 (18.6)	10,112 (16.1)	5,303 (26.3)		
The same as before	32,477 (39.2)	25,147 (40.1)	7,330 (36.4)		
Worsen	34,981 (42.2)	27,459 (43.8)	7,522 (37.3)		
Drinking	8,579 (10.4)	5,904 (9.4)	2,675 (13.3)	244.710	<0.001
Smoking	5,405 (6.5)	3,776 (6.0)	1,629 (8.1)	106.352	<0.001
History of mental disorder	4,755 (5.7)	3,100 (4.9)	1,655 (8.2)	301.308	<0.001

The results of this study showed that COVID-19 pandemic had impacted on students' academic performance and daily life routine. There were 46,980 (56.7%) participants who reported their daily routine had been partly or seriously influenced, and 34,981 (42.2%) participants who reported worsen academic performance.

### 3.2 Stigma toward people with COVID-19

Table 2 shows the comparison of stigma in two groups. 20,155 (24.3%) participants had stigma views toward people infected with COVID-19. The bivariate analysis showed that stigma was significantly associated with male, ethnic minority, obesity, rural area, middle and high school, and monthly family income ≤ 4,999 RMB (*P* < 0.001). For participants with self-infected, family members infected, and being frontline workers, they had significantly lower level of stigma views (*P* < 0.001). Participants who reported willingness to vaccinate during the pandemic or after the restriction lifted had significantly lower level of stigma (*P* < 0.001). Participants with higher level of psychological stress at any stages had significantly higher level of stigma (*P* < 0.001). For participants with history of mental disorder, they had significantly higher level of stigma than those without history of mental disorder (*P* < 0.001).

**Table 2:** The influence factors of stigma views toward COVID-19 patients.

**The influence factors of stigma views toward COVID patients**	**Reference**	**OR**	**95%CI**	***P***
Gender: male	Female	1.397	1.349–1.446	<0.001
Ethnic: Ethnic minority	Han	1.228	1.165–1.294	<0.001
BMI	Normal			
Overweight		0.938	0.882–0.996	0.038
Obesity		1.170	1.127–1.214	<0.001
Household registration: rural	Urban	1.219	1.160–1.281	<0.001
Grade	Middle school			
High school		1.010	0.970–1.052	0.625
College and university		0.804	0.766–0.844	<0.001
Monthly family income	≤4,999			
5,000–19,999		0.961	0.926–0.996	0.031
≥20,000		1.040	0.958–1.130	0.348
Father's education background	Primary school and below			
Middle school		0.961	0.921–1.002	0.064
High school		0.885	0.835–0.937	<0.001
College and above		0.864	0.795–0.939	0.001
Mother's education background	Primary school and below			
Middle school		1.000	0.960–1.041	0.989
High school		1.003	0.946–1.064	0.912
College and above		0.903	0.827–0.987	0.024
Self-infected (confirmed and suspected)	Not	0.872	0.825–0.921	<0.001
Being frontline worker	Not	0.903	0.829–0.983	0.018
Family members infected (confirmed and suspected)	Not	0.718	0.681–0.758	<0.001
Family members had being the frontline workers	Not	0.756	0.683–0.838	<0.001
Vaccination	None			0.026
One does		0.947	0.739–1.213	0.667
Two or more doses		0.820	0.672–1.000	0.050
Willingness to vaccinate during the COVID-19: yes	No	0.812	0.761–0.865	<0.001
Willingness to vaccinate after the restriction lifted: yes	No	0.789	0.755–0.823	<0.001
Psychological stress level during the national lockdown phase: higher	Lower	1.100	1.035–1.168	0.002
Psychological stress level during the regular prevention and control phase: higher	Lower	1.396	1.313–1.485	<0.001
Psychological stress level after the restriction lifted: higher	Lower	1.844	1.767–1.925	<0.001
Quarantine	Not	1.033	0.995–1.073	0.085
Exercise	Not	1.216	1.173–1.260	<0.001
Daily life routine	Not influenced			
Partly influenced		0.787	0.759–0.816	<0.001
Seriously influenced		0.939	0.879–1.002	0.059
Academic performance	Academic progress			
The same as before		0.648	0.620–0.678	<0.001
Worsen		0.610	0.582–0.639	<0.001
Drinking		1.294	1.222–1.370	<0.001
Smoking		1.012	0.944–1.086	0.730
History of mental disorder		1.329	1.243–1.419	<0.001

The Hosmer-Lemeshow test of the model: *P* = 0.75.

### 3.3 The influencing factors of stigma

Table 2 shows the results of logistic regression analysis on the influencing factors of stigma. Being male, ethnic minorities, household registered in rural China, with Body Mass Index (BMI) obesity, exercising during the pandemic, and drinking were risk factors for stigma (OR = 1.397, 1.228, 1.219, 1.170, 1.216, 1.294, *P* < 0.001). Studying in college or university, with BMI overweight, with monthly family income among 5,000–19,999 RMB were protective factors for stigma (OR = 0.804, 0.938, 0.961, *P* < 0.05). In terms of COVID-19-related factors, being self-infected, being a frontline worker, family members being infected, and family members being frontline workers were protective factors for stigma (OR = 0.872, 0.903, 0.718, 0.756, *P* < 0.05). Participants with willingness to vaccinate during the pandemic or after the restriction lifted had significantly lower risk of stigma than those without willingness (*P* < 0.001). Higher level of psychological stress associated with COVID-19 was risk factor for stigma views. For participants with higher level of stress during the national lockdown phase, during the regular prevention and control phase, and after the restriction lifted, the risks of stigma were significantly higher (OR = 1.100, 1.396, 1.844, *P* < 0.001). Compared with participants with academic performance progress, those academic performances remained the same as before or declined had significantly lower risks of stigma (OR = 0.648, 0.610, *P* < 0.001).

### 3.4 The rank of importance of the influence factors

**Figure 1:**
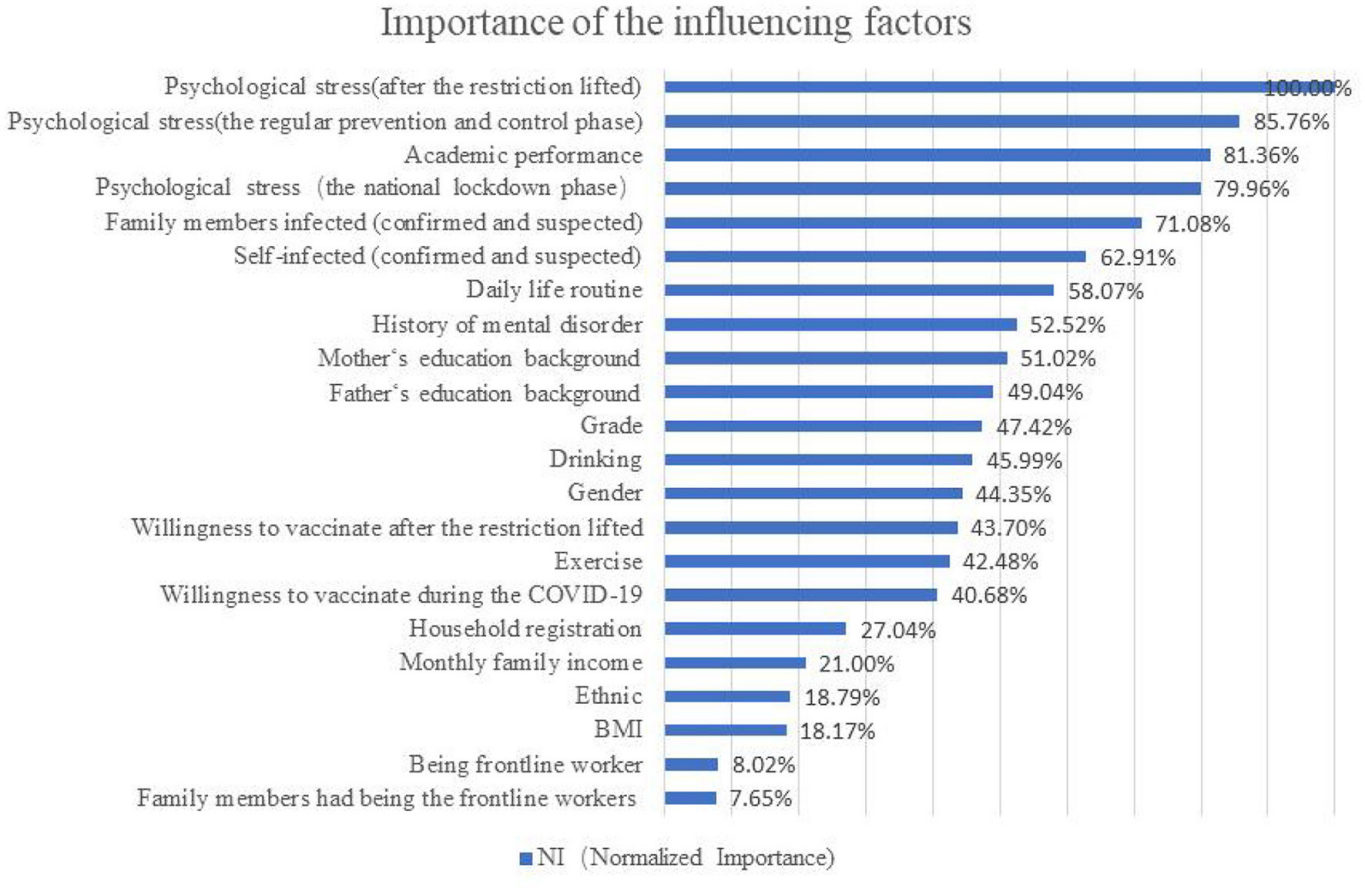
The rank of importance of factors influencing stigma views toward COVID-19 infection.

The results of random forest are shown in Figure 1. The importance of the influencing factors associated with stigma ranged from high to low: psychological stress after the restriction lifted (NI: 100%, NI = Normalized Importance, the most important influencing one was taken as a reference), the psychological stress during the regular prevention and control phase (NI: 85.76%), the impact of COVID-19 pandemic on academic performance (NI: 81.36%), the psychological stress during the national lockdown phase (NI: 79.96%), family members infected with COVID-19 (NI: 71.08%), self-infection (NI: 62.91%), the impact of COVID-19 pandemic on daily life routine (NI: 58.07%), and history of mental disorder (NI: 52.52%).

**Figure 2:**
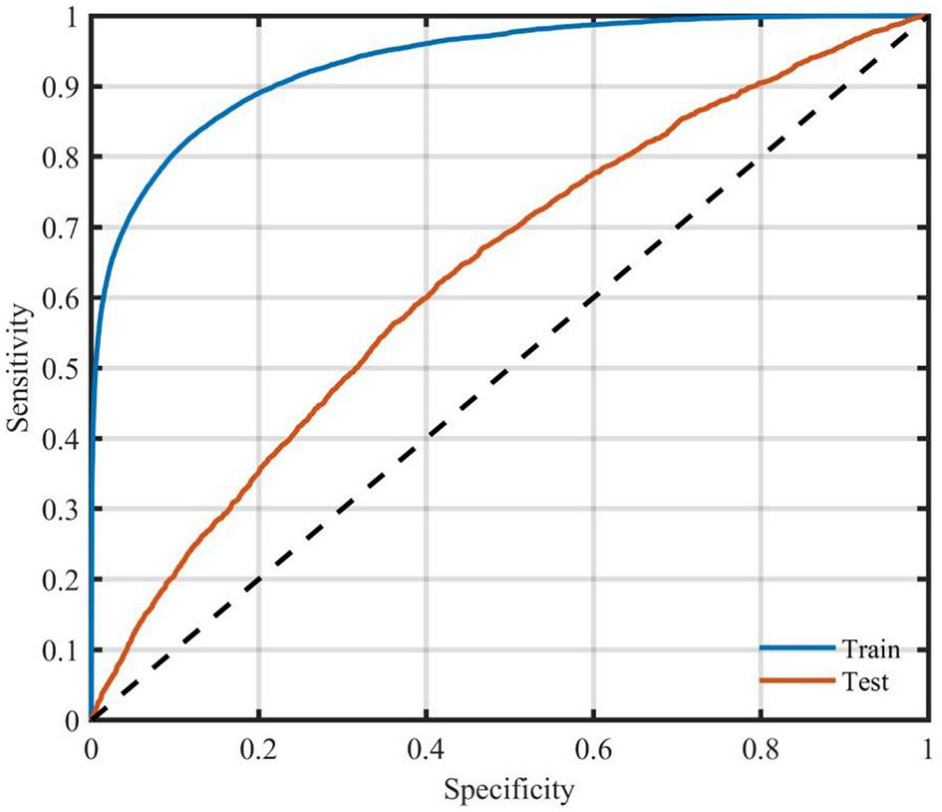
ROC curve of the test sample for the RF model.

There were 66,298 participants (80%) used to train the model, while the remaining 20% (16,575) were include in test set (Table 3). The accuracy of the training model and the test model were 86.99% and 74.90%, respectively. The AUC of ROC of the training and the test model was 0.63 (Figure 2).

**Table 3:** Accuracy rate of RF model.

**Dataset division**	***N***	**Yes**	**No**	**Accuracy rate**
Train set	66,298	57,674	8,624	86.99%
Test set	16,575	12,415	4,160	74.90%

## 4 Discussion

To the best of our knowledge, this is the first study exploring the stigma attitudes toward COVID-19 patients among Chinese adolescent and youth students after the lifting of restriction. Our findings suggest that COVID-19-related stigma among students persists despite improvement in the epidemic situation, and it may have long-term effect on these individuals. Therefore, even after lockdown policy has been lifted, it is essential to explore the stigmatizing attitudes of adolescent and youth students and their influencing factors. Furthermore, this study highlights the importance of factors influencing COVID-19-related stigma, especially those related to disease transmission (e.g., personal and family infection status, psychological stress at various stages etc.).

The prevalence of COVID-19 stigma varies across different countries, different regions within the same country, and even at different stages of the COVID-19 pandemic. This study was conducted in Sichuan Province after the COVID-19 restriction were lifted. The results showed that even after the COVID-19 control measures were lifted, the incidence of stigma toward COVID-19 patients among adolescent and youth students remained as high as 24.3%. A similar study on COVID-19 stigma among Chinese adolescents indicated that its prevalence was 17.2%. This study was carried out from January 2021 to June 2022, in the middle stage of the COVID-19 pandemic in China, and the location was Jingzhou City, Hubei Province, which is not far from Wuhan, the epicenter of the COVID-19 outbreak in China (23). Moreover, there are significant differences in the prevalence of COVID-19 stigma at different countries. For example, in the early stage of the pandemic (May 2020), the prevalence of COVID-19 stigma in China was 31.8% (26). Also, in the early stage of the pandemic, a study in India showed that half (51.3%) of the participants from the community had severe stigmatizing attitudes toward COVID-19 infected individuals (27). Although the prevalence of COVID-19 stigma varies greatly in different contexts, the risk factors of COVID-19 stigma explored in this study are generally consistent with previous researches. Multiple previous studies have shown that male may have stronger COVID-19 stigma (23, 27, 28) and groups with lower monthly family incomes may also exhibit stronger COVID-19 stigma (23).

The rate of stigma among adolescent and youth students toward COVID-19 patients remained high at 24.3% even after the COVID-19 restriction were lifted. The possible reasons maybe as follows. Firstly, this study was conducted during the early phase of the COVID-19 restriction lifted when there was a sharp increase in COVID-19 infections and high risk of infection may led to a high occurrence of stigma. Secondly, the rapid spread of COVID-19 virus is associated with high levels of fear (29), which is positively correlated with COVID-19-related stigma (26, 29). After the restriction were lifted, individuals may have experienced increased fear and anxiety, potentially leading to COVID-19-related stigma and discrimination.

The results of this study indicate that psychological stress during any stages of the COVID-19 pandemic was a significant risk factor for stigma toward COVID-19 patients. In previous studies on stigma, psychological stress has been considered to be closely associated with stigma. Take a study from Israel as an example. This study focused on the group of fathers of adolescents with developmental disabilities. The results showed that there was a significant positive correlation between all types of stress (including marital stress, parenting stress, economic stress, and general stress) and stigma (30). Notably, psychological stress was more severe soon after the lifting of COVID-19 restrictions than during the regular prevention and control phase, or even the national lockdown phase. Several factors may have contributed to this finding. Firstly, during the national lockdown phase, the policy was implemented to curb the spread of virus in the early stages of the pandemic outbreak. However, in non-epicenter areas such as Sichuan province, people's understanding of the virus's risks and the extent of the spread may be limited compared to the latter two stages. As a result, people may have perceived a lower risk of COVID-19 infection, minimizing the impact of psychological stress during this phase. Secondly, a previous study showed that people living high case areas were more likely to stigmatize to persons with COVID-19 infection (28). After the lifting of the COVID-19 restrictions, there was a sharp increase in infections, and people faced heightened risk of infection. This increased risk may have led to a greater sense of threat, resulting in higher levels of psychological stress during this stage. Furthermore, our findings suggest that the impact of psychological stress on COVID-19-related stigma was more significant than the impact of COVID-19 infection itself at any stage. This highlights the importance of addressing psychological stress in mitigating mental health outcomes, consistent with previous study (20).

The results of this study found that stigma views were negatively associated with both self-infection and family members' infection, with the latter having a more significant impact on the stigma. In contrast, previous research has indicated that being a recovered COVID-19 patient or having family members infected with COVID-19 is positively correlated with a higher overall sense of stigma (31). This difference may be related to the stage of the pandemic. In the middle stage of the pandemic, the number of COVID-19 infected people was relatively small, and people had limited information and knowledge about COVID-19. Therefore, they were more likely to hold stigmatizing attitudes toward COVID-19 patients (28). This study was conducted after the COVID-19 restriction were lifted. At this time, there were a large number of COVID-19 infected people. After personal or family member infections, people gained a deeper understanding of the disease, which led to a decrease in stigma. Moreover, several factors may also contribute to this finding. Firstly, stigma is a phenomenon of social distinction and labeling, where individuals with a perceived attribute (e.g., COVID-19 infection) are categorized as “them” and separated from “us” (4). However, when individuals themselves are infected, the formation of stigma toward COVID-19 patients (including themselves) may be disrupted. Secondly, previous studies have shown that household transmission was the primary mode of COVID-19 growth in China (32–34) It was challenging for individuals to wear masks or take other protective measures before their family members were confirmed or suspected to be infected (34), and others may view them as sources of infection, thereby reducing the risk of stigma. Thirdly, when individuals or their family members are confirmed or suspected to be infected with COVID-19, they may change their stigma attitude to resolve cognitive dissonance (35), reflecting the importance of family notion in traditional Chinese culture. Furthermore, personal experience with infection may increase COVID-19-related knowledge and reduce stigma risk. Notably, our study also found that being a frontline worker or having a family member as a frontline worker was a protective factor against stigma, suggesting that contact with infected individuals may reduce stigma views. This finding has implications for the development of anti-stigma interventions, which could involve increasing social contact with persons infected with COVID-19. By promoting understanding and empathy through social interaction, such interventions may help mitigate stigma and promote a more supportive social environment.

The results of this study showed that the academic performance was the third most influential factor associated with stigma toward COVID-19 patients. The impact of COVID-19 pandemic on students' academic performance significantly influenced their stigma views toward the pandemic and infected individuals. Notably, students who experienced academic performance progress during the COVID-19 pandemic had a significantly higher risk of stigma compared to those whose academic performance declined or remained the same. Further longitudinal studies should be conducted to explore the long-term impact in this area.

Consistent with previous studies (26, 28, 36, 37), our results showed that age was positively associated with stigma. The risk of COVID-19-related stigma varied across different learning stages or ages, with high school students exhibiting significantly higher risks compared to middle school and college students. This finding is in line with a previous study that reported lower stigma risks among students aged 18 and above compared to those aged 15–17 (23). Students in high school might face greater academic pressure, and the pandemic's impact on their studies and future prospects may be more pronounced. In contrast, middle school students were more susceptible to prejudice due to limited assess to health information and lower health knowledge about COVID-19.

Research found that stigma and discrimination were associated with sociodemographic factors, including gender, ethnicity, and social class (5, 23, 28). Male students were at a higher risk of stigma toward COVID-19 patients than female students, consistent with previous studies (23, 28, 38). Additionally, ethnic minority students, those with rural household registrations, and those from families with lower educational background, and monthly incomes <4,999 RMB were more likely to exhibit stigma. This may be attributed to the limited social resources available to these students and their families. Previous research has shown that economically disadvantaged individuals with insufficient education may be more likely to hold stigma views (28). Limited social resources (e.g., lack of knowledge, poor economic conditions) may hinder coping mechanisms and increase the risk of stigma (9). Therefore, it is crucial to develop health policies, interventions and social resources that improve social support and health knowledge, especially for minority ethnic individuals, to prevent stigmatization.

Based on the above discussion, the research findings offer significant guidance for policy-making. First, the timing of lifting restrictions on infectious diseases is key for stigma intervention. Health authorities should quickly share accurate post-lockdown infection information via various channels to help adolescents rationalize and reduce stigma from fear. Schools can integrate short-term COVID-19 modules into the curriculum in different forms, enabling students to develop a correct understanding of the disease, and adopt a non-discriminatory attitude toward patients. Second, this study indicates that there is a strong connection between psychological stress and the stigma associated with infectious diseases. Therefore, when conducting stigma intervention, it is particularly important to simultaneously address psychological stress. Third, the findings indicate that there is a negative correlation between self or family members' COVID-19 infection and stigma, and being a frontline worker is a protective factor against stigma. This may suggest that encouraging the public to serve as volunteers is one of the effective solutions for stigma intervention. Relevant intervention programs can be established based on this, increasing the public's contact with patients, helping them understand the disease correctly, and reducing the public's stigma. Fourth, the research shows that there are differences in the stigma of infectious diseases among genders, age groups, academic performance levels, and economic statuses. Subsequent interventions can formulate targeted plans according to these different situations.

### 4.1 Strengths and limitations

This study analyzed over 80,000 adolescents and college students, which provides strong statistical power and allows for robust multivariate analyses. Also, this study focuses on a timely and crucial topic: the prevalence of COVID-19 - related stigma among young students after the lifting of pandemic restrictions, addressing an area that is vital for the well being of this vulnerable population. Using both logistic regression analysis and Random Forest (RF) modeling, this study shows that higher levels of psychological stress, particularly post-restriction lifting, significant emerged as a risk factor for stigma. Both self-infection and family members' infection were risk factors of stigma, although psychological stress proved more influential.

Due to some limitations, the results of this study should be explained carefully. First, this study was a cross-sectional study using a self-rating scale, which cannot infer causality. The self-rating scale may produce biased results due to differences in the cultural level and intelligence level of the participants. Future research should use longitudinal or prospective designs to clarify the causal and temporal relationships between stigma development and influencing variables, as well as triangulate self-report data with other sources such as peer ratings or behavioral indicators to reduce bias and validate findings. Second, the sample of this study was only from Sichuan Province, China, likely not representing students in other regions with different cultural, economic, or pandemic-related contexts. Future studies should include participants from multiple regions or diverse backgrounds to enhance findings' generalizability and applicability. Third, although this study has taken into account numerous demographic and social factors, there are still some potential key variables that have been overlooked. The analysis didn't consider potentially influential variables like health literacy, media exposure, source credibility, and direct contact with infected individuals, which may impact stigma. Future studies should integrate more psychosocial and informational variables for a comprehensive stigma-formation model. Fourth, this study only employed the random forest method, which has certain limitations. In future predictive analyses, it is necessary to further optimize the model, explore more algorithms or ensemble methods, and combine feature engineering techniques to improve the classification accuracy.

## 5 Conclusion

The COVID-19 pandemic's impact on mental health will persist in the long term. Even after the lifting of restrictions such as quarantine and lockdown, severe stigma toward COVID-19 infected individuals remain prevalent among youth students. Our study identified several key risk factors for stigma, including male sex, high school students, and the impact of COVID-19 on academic performance. Additionally, students with limited resources (e.g., ethnic minorities, rural households, parents with lower educational attainment) were more likely to exhibit stigma. Higher levels of psychological stress at various study stages also emerged as a significant risk factor for stigma. Conversely, self-infection and close contact with infected individuals, such as family members or through frontline work, were found to be protective factor against stigma. However, our analysis revealed that psychological stress had a more profound impact on stigma than infection or contact with infected individuals. These findings underscore the need for targeted health policies and interventions to mitigate COVID-19-related stigma and promote the mental health and well being of youth students.

## Data Availability

The raw data supporting the conclusions of this article will be made available by the authors, without undue reservation.
